# Transient receptor potential vanilloid 4 mediates sour taste sensing via type III taste cell differentiation

**DOI:** 10.1038/s41598-019-43254-y

**Published:** 2019-04-30

**Authors:** Kenjiro Matsumoto, Akihiro Ohishi, Ken Iwatsuki, Kaho Yamazaki, Satoko Takayanagi, Masahiro Tsuji, Eitaro Aihara, Daichi Utsumi, Takuya Tsukahara, Makoto Tominaga, Kazuki Nagasawa, Shinichi Kato

**Affiliations:** 1 Division of Pathological Sciences, Department of Pharmacology and Experimental Therapeutics, Kyoto Pharmaceutical University, Kyoto, Japan; 2 Division of Biological Sciences, Department of Environmental Biochemistry, Kyoto Pharmaceutical University, Kyoto, Japan; 3 Department of Nutritional Science and Food Safety, Faculty of Applied Bioscience, Tokyo University of Agriculture, Tokyo, Japan; 4 Department of Pharmacology and Systems Physiology, University of Cincinnati, Cincinnati, Ohio USA; 5 Division of Cell Signaling, Okazaki Institute for Integrative Bioscience (National Institute for Physiological Sciences), Okazaki, Japan

**Keywords:** Developmental biology, Physiology

## Abstract

Taste buds are comprised of taste cells, which are classified into types I to IV. Transient receptor potential (TRP) channels play a significant role in taste perception. TRP vanilloid 4 (TRPV4) is a non-selective cation channel that responds to mechanical, thermal, and chemical stimuli. The present study aimed to define the function and expression of TRPV4 in taste buds using *Trpv4*-deficient mice. In circumvallate papillae, TRPV4 colocalized with a type IV cell and epithelial cell marker but not type I, II, or III markers. Behavioural studies showed that *Trpv4* deficiency reduced sensitivity to sourness but not to sweet, umami, salty, and bitter tastes. *Trpv4* deficiency significantly reduced the expression of type III cells compared with that in wild type (WT) mice *in vivo* and in taste bud organoid experiments. *Trpv4* deficiency also significantly reduced Ki67-positive cells and β-catenin expression compared with those in WT circumvallate papillae. Together, the present results suggest that TRPV4 contributes to sour taste sensing by regulating type III taste cell differentiation in mice.

## Introduction

We sense five basic tastes (bitter, sweet, umami, salty, and sour) across all regions of the tongue where taste buds exist. Taste buds are comprised of taste cells, which are classified into types I to IV^1^. Type I cells are generally thought to have a support function in the taste bud^2^. Type II cells detect sweet, bitter, and umami tastants via a common intracellular transduction cascade including PLCβ2^3^. Type III taste cells are sour detectors and respond to acid taste stimulation by releasing serotonin^4,5^. The cell type mediating salty taste remains ambiguous^6^. Type IV basal cells differentiate into type I, II, and III taste cells during rapid cell turnover in taste buds^2^. Taste afferent nerve fibres transmit information from taste buds to the brain^7^.

Transient receptor potential (TRP) channels are non-selective cation channels activated by a variety of chemical and physical stimuli, such as temperature, oxidative stress, and osmotic pressure, and by food-derived products, such as capsaicin, menthol, and various lipids^8^. Proteins related to TRP channels, including TRP-melastatin 5 (TRPM5), polycystic kidney disease-1-like 3 (PKD1L3), and polycystic kidney disease-2-like 1 (PKD2L1), are also expressed in taste cells^9^. It is thus possible that these TRP channels play some roles in taste sensing^10^. In fact, TRPM5 has a well-defined role in the detection of bitter, sweet, and umami stimuli and is exclusively expressed in type II taste cells^11^.

TRPV4 is a non-selective cation channel that responds to mechanical, thermal, and chemical stimuli in addition to various endogenous ligands, such as arachidonic acid metabolites^12^. We recently reported that TRPV4 was expressed in the mouse gastrointestinal tract, where it regulated pathophysiological functions^13^. Furthermore, several studies have demonstrated possible mechano- and osmosensing roles of TRPV4 in taste buds^14,15^. TPRV4 is reportedly localized in sensory neurons^16,17^ and epithelial cells but not in taste buds of the tongue^15^. In contrast, TRPV4 immunostaining was observed in zebrafish taste buds^18^. At this stage, the localization and implied functions of TRPV4 in the taste buds and in taste sensation remain undefined. The present study aimed to define the expression of TRPV4 in the taste buds and investigate the roles of TRPV4 in taste perception using *Trpv4*-deficient mice.

## Results

### TRPV4 immunoreactivity is detected in type IV taste cells and epithelial cells of mouse circumvallate papillae

**Figure 1:**
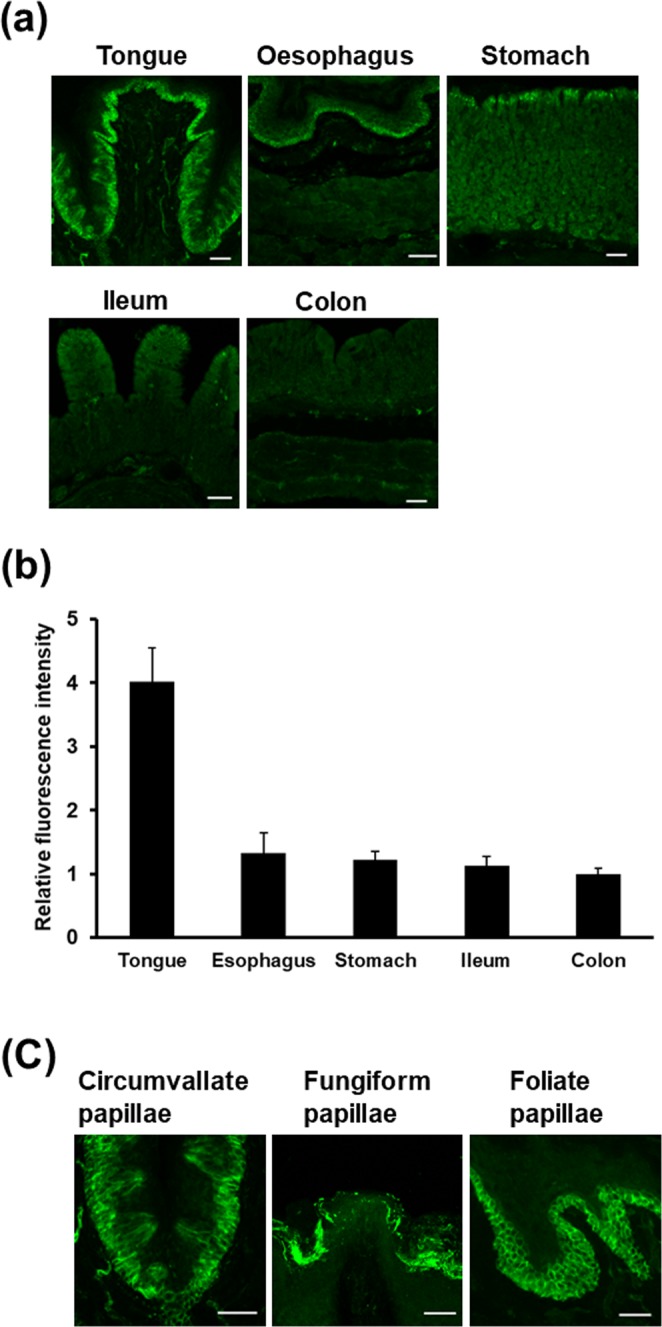
Expression of TRPV4 in mouse tissues. (**a**) Immunohistochemistry of TRPV4 in the tongue, oesophagus, stomach, ileum, and colon. (**b**) Quantitative analysis of TRPV4 expression in the tongue, oesophagus, stomach, ileum, and colon. Data presented as the means ± SEM for 8 mice. (**c**) TRPV4 expression in the circumvallate, fungiform, and foliate papillae. Scale bars = 50 μm.

First, we investigated the expression of TRPV4 in the mouse tongue, oesophagus, stomach, ileum, and colon by immunohistochemistry (Fig. 1a). TRPV4 immunoreactivity was detected in epithelial-like cells of the mouse tongue, oesophagus, stomach, ileum, and colon. Within the mouse tongue, oesophagus, stomach, ileum, and colon, TRPV4 expression was highest in the tongue according to fluorescence intensities (Fig. 1b). We also found TRPV4 expression in the mouse circumvallate, fungiform, and foliate papillae (Fig. 1c).

**Figure 2:**
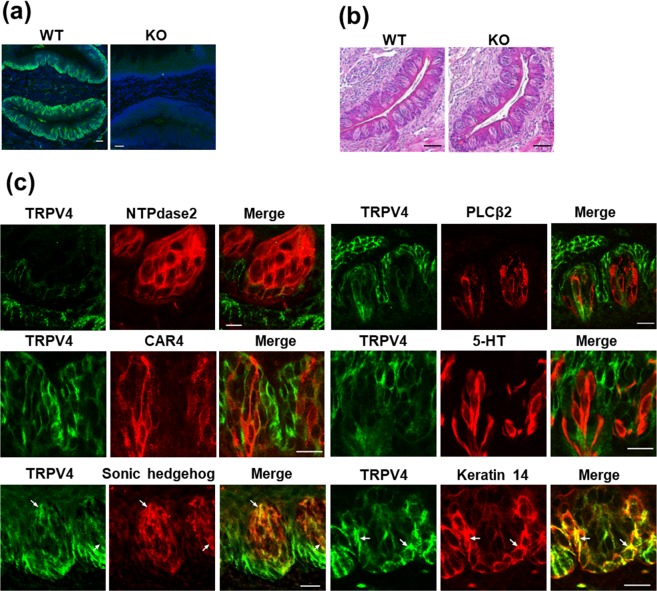
TRPV4 expression in WT and *Trpv4* KO mice. (**a**) TRPV4 expression in the circumvallate papillae. (**b**) Representative images of haematoxylin and eosin staining of WT and *Trpv4* KO. (**c**) Double labelling experiments of TRPV4 (green) with NTPdase2, PLCβ2, CAR4, 5-HT, Sonic hedgehog, and keratin 14 (red) in the circumvallate papillae. Scale bars = 50 μm (**a**,**b**) and 20 μm (**c**). Arrows indicate colocalization of signals of TRPV4 and keratin 14.

TRPV4 immunoreactivity in the circumvallate papillae was abolished in *Trpv4* knockout (KO) mice (Fig. 2a). There were no structural differences between wild type (WT) and *Trpv4* KO circumvallate papillae (Fig. 2b). Double-labelling experiments of TRPV4 with the type I taste cell marker NTPdase2, type II taste cell marker PLCβ2, type III taste cell markers CAR4 and 5-HT, type IV taste cell marker Sonic hedgehog, and basal keratinocyte marker keratin 14 were performed in circumvallate papillae (Fig. 2c). TRPV4 colocalized with Sonic hedgehog and keratin 14 but did not colocalize with any of the other markers.

### *Trpv4*-deficient mice exhibit decreased behavioural response to sour tastants

**Figure 3:**
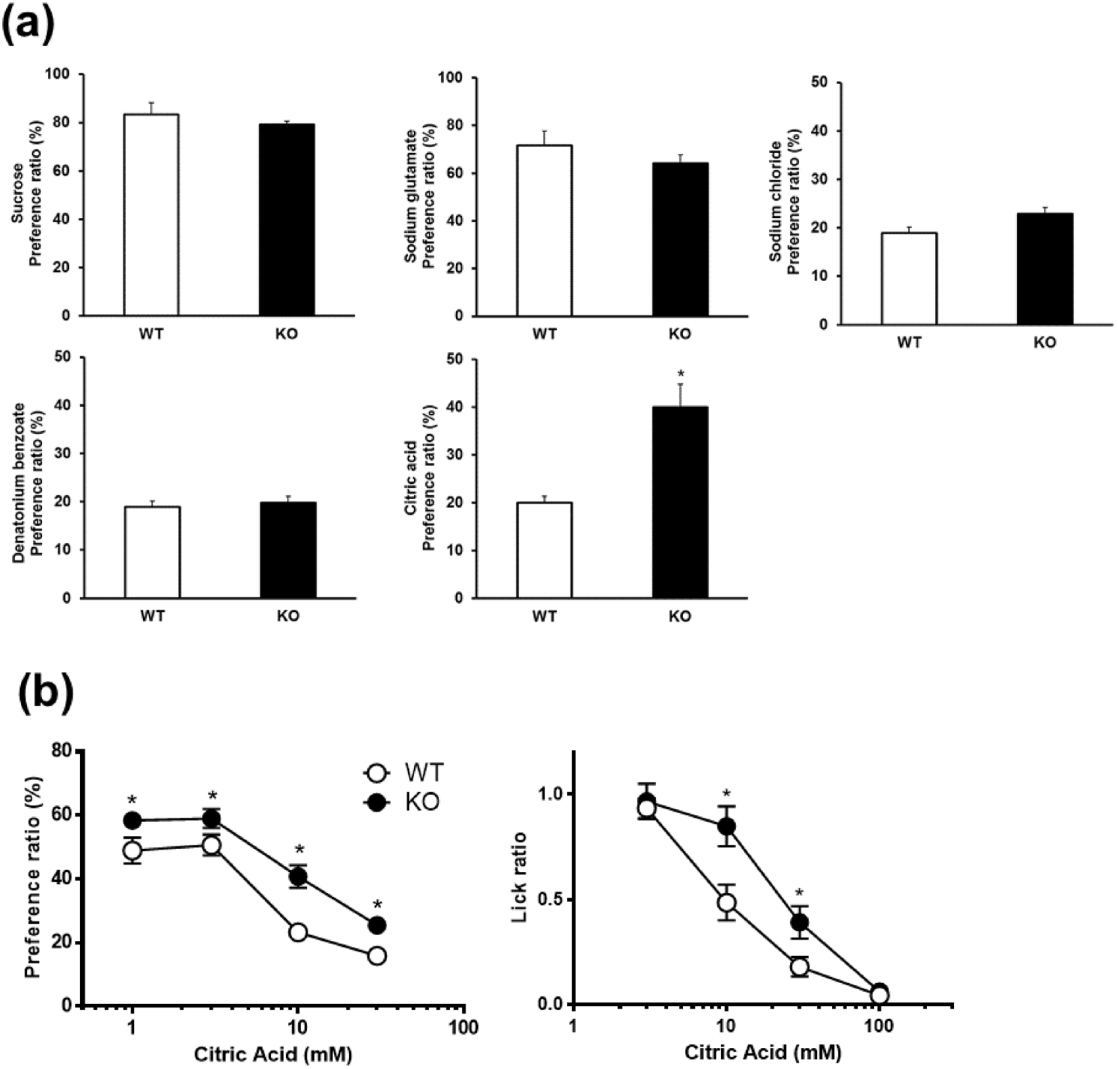
Behavioural responses of WT and *Trpv4* KO mice to sour tastants. (**a**) Two-bottle tests were used to evaluate behavioural responses to sweet (sucrose, 30 mM), umami (monosodium glutamate, 100 mM), bitter (denatonium benzoate, 1 mM), salty (sodium chloride, 300 mM), and sour taste (citric acid, 10 mM) stimuli. Data presented as the means ± SEM for 8 mice. For statistical analysis, Student’s *t*-test was used. *P < 0.05 vs. WT. (**b**) Two-bottle (left) and brief-access (right) tests were used to measure behavioural responses to sour taste stimuli. Data presented as the means ± SEM for 8 mice. For statistical analysis, two-way ANOVA followed by Bonferroni’s multiple comparison test was used. *P < 0.05 vs. WT.

To determine the taste sensitivities of *Trpv4* KO mice, we performed a two-bottle test (Fig. 3a). WT and *Trpv4* KO mice were subjected to a preference test between water and an appetitive concentration of sucrose and sodium glutamate, followed by an aversion test between water and an aversive concentration of sodium chloride and denatonium benzoate. *Trpv4* KO mice showed diminished avoidance of the citric acid solution compared with that of WT mice.

To assess the change in sour taste sensitivity in more detail, we performed two-bottle and brief-access tests using different concentrations of citric acid solutions (Fig. 3b). A statistical analysis by two-way analysis of variance (ANOVA) showed a significant difference in sour taste sensitivity between WT and *Trpv4* KO mice for both tests (two-bottle test: *p* = 0.0009, *F*_(1, 12)_ = 19.39; brief-access test: *p* = 0.014, *F*_(1, 14)_ = 7.868). A post-hoc comparison test showed that the preference ratios in the *Trpv4* KO mice for the 1, 3, 10, and 30 mM citric acid solutions were significantly higher than those in WT mice in the two-bottle test. Similarly, for the brief-access test, a post-hoc comparison test showed that the lick ratios in the *Trpv4* KO mice for the 10 and 30 mM citric acid solutions were significantly lower than those in WT mice.

### *Trpv4* deficiency reduces type III taste marker expression in circumvallate papillae

**Figure 4:**
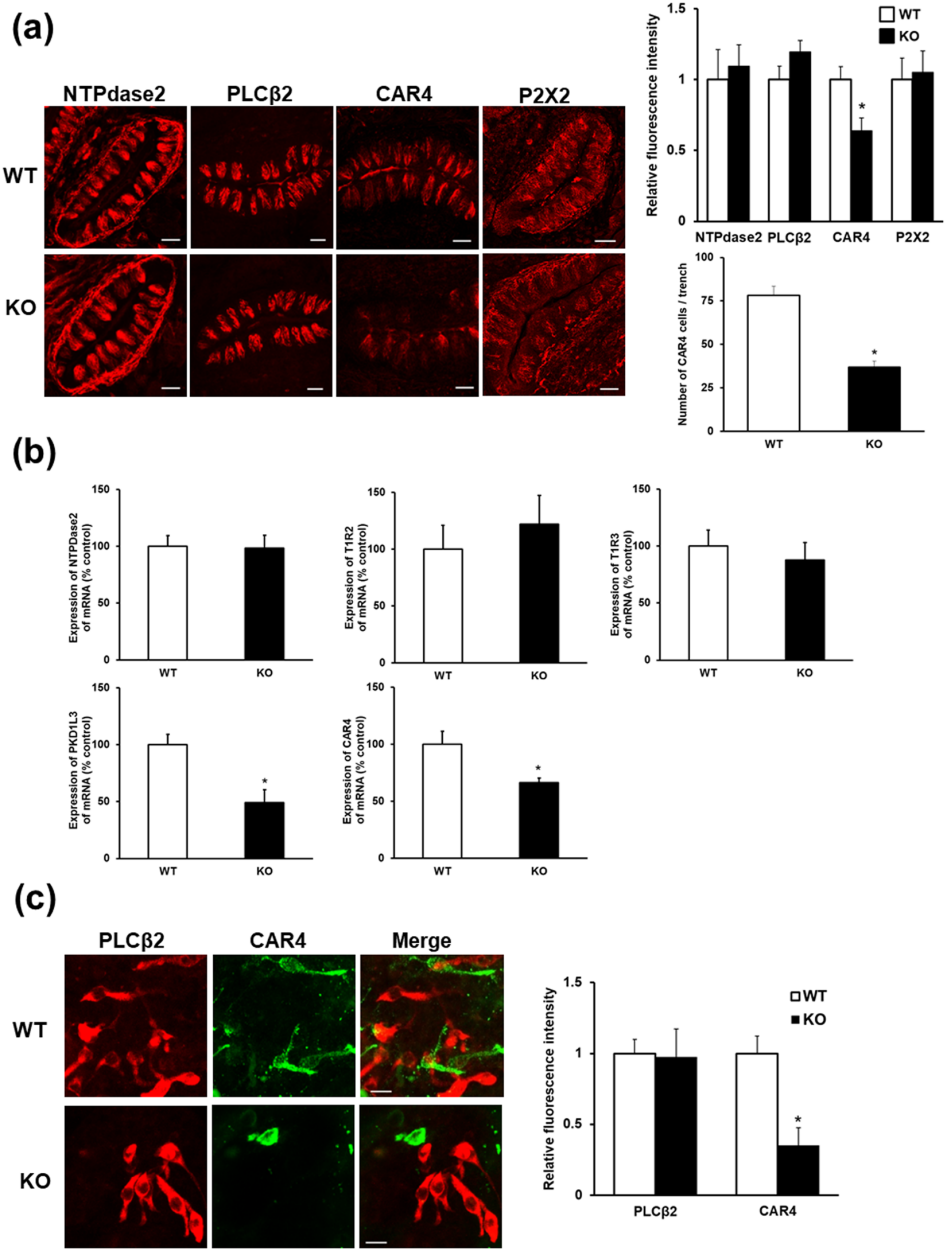
Type III taste cell marker expression in circumvallate papillae and taste organoids of WT and *Trpv4* KO mice. (**a**) Representative images and quantitative analysis of NTPdase2, PLCβ2, CAR4, and P2X2 expression in circumvallate papillae. (**b**) Quantitative analysis of *NTPdase2*, *T1r2*, *T1r3*, *Pkd1l3*, and *CAR4* mRNA expression in circumvallate papillae. (**c**) Representative images and quantitative analysis of PLCβ2 and CAR4 expression in taste organoids derived from WT and *Trpv4* KO. A taste organoid was derived from each WT or *Trpv4* KO mouse. Data are presented as the mean ± SEM for 6–8 mice. For statistical analysis, Student’s *t*-test was used. *P < 0.05 vs. WT.

In mice, taste buds are composed of collections of type I, II, and III taste cells. We next assessed changes in type I, II, and III taste cells and taste nerve cells in *Trpv4* KO mice using NTPdase2, PLCβ2, CAR4, and P2X2 as markers. There was no significant difference in the fluorescence intensity of NTPdase2, PLCβ2, or P2X2 between WT and *Trpv4* KO mice (Fig. 4a). In contrast, the fluorescence intensity and cell numbers of CAR4 in *Trpv4* KO mouse taste buds were significantly reduced compared to those in WT mice.

Next, we investigated the mRNA expression levels of *NTPdase2*, *T1r2*, *T1r3*, *Pkd1l3*, and *CAR4* in WT and *Trpv4* KO mice (Fig. 4b). Consistent with the immunofluorescence results, the mRNA expression levels of the type III markers *Pkd1l3* and *CAR4* in the circumvallate papillae of *Trpv4* KO mice were significantly reduced compared to WT levels. There was no significant difference in the expression levels of the type I taste cell marker *NTPdase2* or type II taste cell markers *T1r2* and *T1r3* between WT and *Trpv4* KO mice.

Next, the effect of *Trpv4* ablation on type III taste cell differentiation was tested in taste organoids derived from WT and *Trpv4* KO circumvallate papillae (Fig. 4c). Immunostaining of *Trpv4* KO organoids showed that the expression level of CAR4-immunopositive cells was significantly reduced compared with that in WT organoids, while that of PLCβ2-immunopositive cells was unchanged.

### *Trpv4* deficiency affects the expression of β-catenin in circumvallate papillae

**Figure 5:**
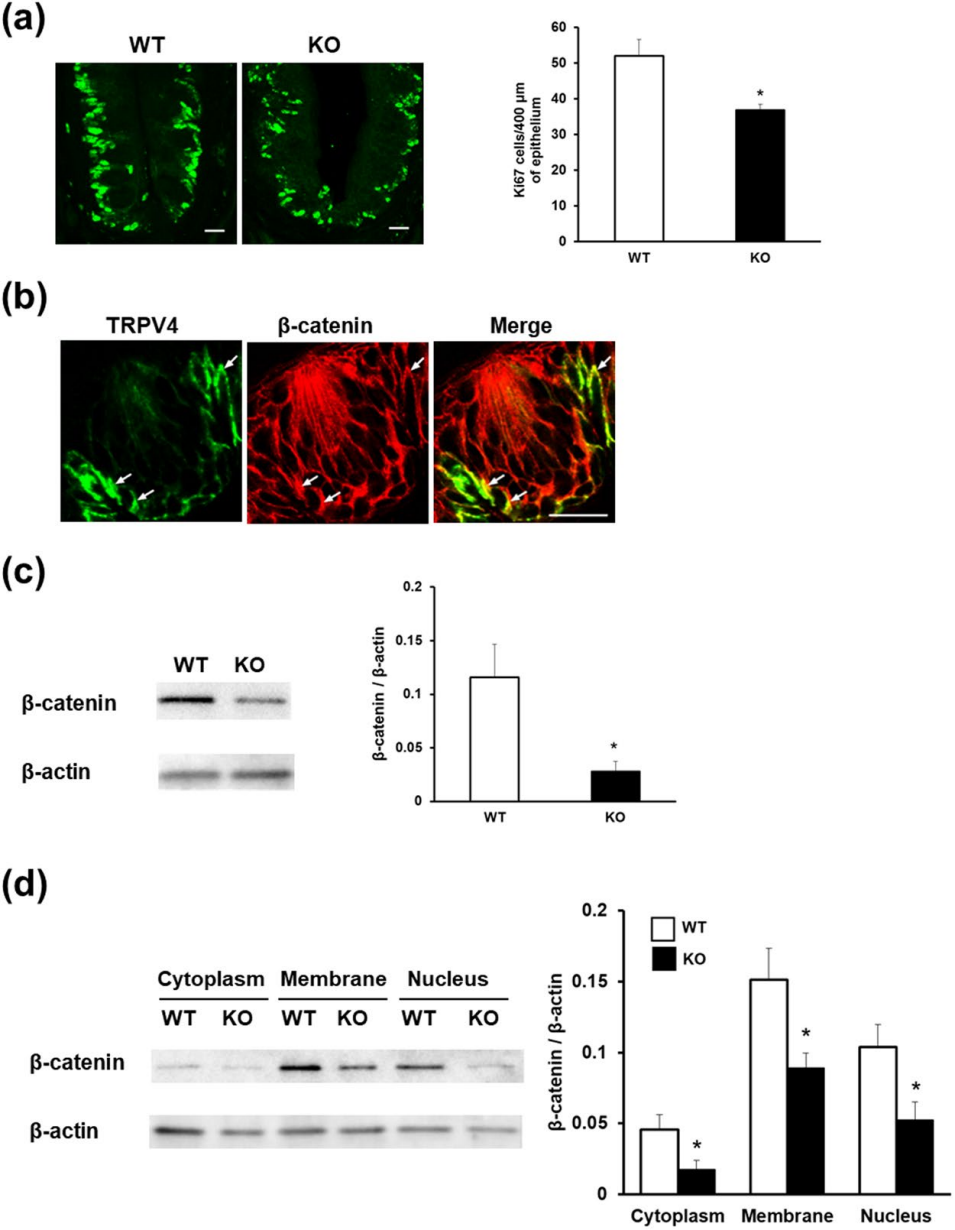
Effect of *Trpv4* deficiency on Ki67 and β-catenin expression in circumvallate papillae and analysis of TRPV4 expression in circumvallate papillae subfractions. (**a**) Number of Ki67-immunopositive basal cells per 400 μm^2^ of epithelium in the circumvallate papillae of WT and *Trpv4* KO mice. (**b**) Double-labelling of TRPV4 (green) and β-catenin (red) in circumvallate papillae. (**c**) β-catenin expression in circumvallate papillae of WT and *Trpv4* KO mice. (**d**) β-catenin expression in cytoplasmic, membrane, and nuclear fractions of circumvallate papillae. Data are presented as the mean ± SEM for 8 mice. For statistical analysis, Student’s *t*-test was used. *P < 0.05 vs. WT.

To investigate the role of TRPV4 in the differentiation of taste cells, we compared the proportions of Ki67 and β-catenin expression in WT and *Trpv4* KO mice (Fig. 5). Quantitative analysis showed that proliferating cell marker Ki67 was significantly decreased in the circumvallate papillae of *Trpv4* KO mice compared with that in WT mice (Fig. 5a). β-catenin is a representative key regulator of taste cell renewal in mice. TRPV4 immunoreactivity in basal cells colocalized with that of β-catenin in circumvallate papillae (Fig. 5b). Western blotting analysis showed that expression of β-catenin was significantly reduced in *Trpv4* KO mice compared with that WT mice (Fig. 5c). Finally, we performed subcellular fractionation of protein lysates and analysed cytosolic, membrane, and nuclear fractions by immunoblotting for β-catenin proteins in WT and *Trpv4* KO mouse circumvallate papillae (Fig. 5d). The expression of β-catenin in all fractions was significantly lower in *Trpv4* KO mice than in WT mice.

## Discussion

The sense of taste is responsible for the detection of bitter, sweet, umami, salty, and sour tastants by taste buds. TRP channels play an important role in taste, with channels responding to basic tastants. The current study demonstrated for the first time that TRPV4 is expressed in type IV cells of the taste buds. *Trpv4* deficiency significantly reduced the sensitivity of sourness and the expression of type III cells compared with those in WT mice. These findings suggest that TRPV4 contributes to sour taste sensing by regulating type III taste cell differentiation in mice.

Previous studies have detected TRPV4 immunoreactivity in the epithelial cells of the mammalian alimentary tract^13,19,20^. We also detected TRPV4 immunoreactivity in epithelial cell-like structures in the gastrointestinal tract. Within the mouse tongue, oesophagus, stomach, ileum, and colon, TRPV4 expression was highest in the tongue. We further observed the expression of TRPV4 in circumvallate, fungiform, and foliate papillae. Circumvallate papillae, located on the surfaces of the back part of the tongue, contain higher numbers of taste buds than other papillae. It is thus likely that TRPV4 expressed on the oral side, in the circumvallate papillae, plays a role in the taste function.

TRPV4-immunopositive cells were previously identified in zebrafish taste buds^18^. In contrast, TRPV4 was found to be expressed in the epithelial cells but not the taste buds of the mouse tongue^15^. In the present study, we observed TRPV4 immunoreactivity in Sonic hedgehog-positive type IV cells in mouse circumvallate papillae. However, TRPV4 immunoreactivity did not colocalize with type I, II, and III taste cell markers. Sonic hedgehog-positive cells are precursors for all taste cell types within taste buds^21^. It has been previously reported that TRPV4 is expressed in keratin 14-positive epidermal keratinocytes and in the olfactory and airway epithelia^22,23^. In this study, we also observed TRPV4 immunoreactivity in keratin 14-positive basal epithelial cells. Keratin 14-positive epithelial cells located outside of the taste bud are bipotential progenitor cells that can generate both taste bud cells and keratinocytes^24^. These results suggest that TRPV4 is expressed in type IV cells and keratin 14-positive epithelial cells in the circumvallate papillae.

Some TRP channels are expressed in taste buds and have functional roles as detectors of taste. TRPM5 is known to be abundantly expressed in type II taste cells, where it participates in sweet, bitter, and umami taste perception^25,26^. In the present study, we found that *Trpv4* deficiency reduced the sensitivity to sour taste but not to sweet, umami, salty, or bitter tastes in two-bottle tests. As previously reported, TRPV4 agonist 4α-phorbol 12,13-didecanoate administration did not affect the intake of sucrose solution^27^. Consequently, we compared the behaviour of *Trpv4* KO and WT mice using different concentrations of citric acid solution presented in brief-access and two-bottle tests. *Trpv4* deficiency tended to reduce taste aversion to sourness in both tests. These results imply that TRPV4 has an important role in sour taste sensing in mice. Type III cells respond directly to a variety of acids, such as citric acid, acetic acid, and HCl, suggesting that type III cells are involved in sour taste sensing^4,5,28^. Basal cells differentiate into new type I, II, and/or III taste cells during cell turnover^2^. Presynaptic type III taste cells survive for approximately one month, while type II taste cells have a life span of about one week and type I taste cells survive for 2–3 days^29,30^. Sour compounds are detected by type III taste cells, which represent a small population of cells within taste buds (~15%)^4,31^. The activation of type III taste cells in mammalian taste buds is implicated in the transduction of sour taste. To investigate the relationship between TRPV4 and sour taste detection, we compared the expression of type III taste cell markers between WT and *Trpv4* KO mice. PKD2L1 and PKD1L3 may serve as sour taste sensors^28,32^. *Trpv4* deficiency significantly reduced the mRNA levels of *PKD1L3* and *CAR4* and protein expression levels of the type III cell marker but did not affect type I or II cell marker expression levels. Taste bud organoids derived from circumvallate papillae can stably express differentiated cell types specific to the native organ^33^. Because taste organoids are not influenced by signals from other tissues, the results of genetic or other manipulations can be interpreted in a more straightforward manner^33,34^. The effect of *Trpv4* deficiency in taste organoids reproduced the observations of *in vivo* experiments involving PLCβ2 and CAR4 expression in taste buds. These results suggest that TRPV4 regulates type III taste cell differentiation without affecting type I or II taste cells in mouse taste buds.

In brief-access and two-bottle tests, *Trpv4* deficiency did not completely diminish taste aversion to sourness. Purinergic signalling, including P2X2 and/or P2X3 expressed on taste nerves, also contributes to taste sensing. Indeed, several studies have demonstrated that mice lacking P2X2 and P2X3 receptors exhibit essentially no response to all classes of tastants, including acids^35,36^. In the present study, *Trpv4* deficiency did not affect the expression levels of taste nerve markers (P2X2 receptor) compared with those in WT. In contrast, *Trpv4* deficiency significantly reduced the expression levels of type III cell markers (CAR4) compared with those in WT but did not completely abolish expression. Thus, it is likely that the remaining sour taste aversion in *Trpv4* KO mice may be based on sour sensing via taste nerves and remaining type III taste cells.

Type III cells may also respond to high concentrations of NaCl^37^. A previous study also showed amiloride-insensitive salt taste-like responses in a subset of bitter-sensitive type II cells^38^. Based on these findings, the relationship between salty taste detection and TRPV4 is unclear at present, although it is possible that TRPV4 affects the type III taste cell function and/or expression.

Wnt/β-catenin signalling is involved in taste cell fate determination^38,39^. β-catenin activates the transcription of target genes in type IV basal cells to induce their differentiation into type I, II, and/or III cells. A previous study reported that TRPV4 interacts with ß-catenin at adherens junctions in keratinocytes^40^ and in the membranes of endothelial cells^41^. We found that TRPV4 colocalized with β-catenin in the basal cells of taste buds. Furthermore, *Trpv4* deficiency significantly reduced β-catenin expression in cytosolic, membrane, and nuclear fractions. In endothelial cells, shear stress stimulation disrupted the interaction of TRPV4 with β-catenin in the basal membrane^42^. From these results, we speculate that *Trpv4* deletion interrupts the interaction with β-catenin in the basal cell membrane and affects β-catenin expression in the taste buds. Conditional β-catenin deletion in mouse taste progenitor cells leads to taste bud loss and alterations in behavioural taste sensitivity^43^. This mutant also exhibited reductions in type I, II, and III taste cells. Conditional β-catenin deletion significantly reduced the perception of artificial sweetener SC45647 (sweet), denatonium (bitter), and a low concentration (1 mM) of citric acid (sour) but did not affect perception of high concentrations (30 and 100 mM) of citric acid. In the present study, we found that *Trpv4* deficiency significantly reduced selective type III taste cells and citric acid (1 to 30 mM) perception compared with those of WT mice. However, the mechanism for the selective regulation of type III taste cells remains unclear. Further studies are needed to clarify the involvement of TRPV4 in regulating β-catenin expression and type III taste cell differentiation in circumvallate papillae. Additional molecular factors likely contribute to the regulation of the fate of type IV basal cells. The Sonic hedgehog and Notch pathways are also thought to be involved in taste cell fate determination^2,34^. Thus, further investigations of the relationship between TRPV4 and these other molecular factors are needed.

In conclusion, TRPV4 is involved in sour taste perception through the differentiation of type III taste cells via the regulation of β-catenin expression. Our results imply that TRPV4 is a key molecule in the differentiation of sour taste cells in mammalian taste bud stem cells.

## Methods

### Animals

Male C57BL/6 mice (8–10 weeks) weighing 22–27 g were purchased from Japan SLC Inc. (Shizuoka, Japan). *Trpv4* KO mice were generated in a C57BL/6J background as described previously^15^. As previously reported, female hormones affect behavioural taste sensitivity in mice^41^. Therefore, we used only male mice in this study. All mice were maintained in plastic cages with free access to food and water and housed at 22 ± 1 °C under a 12-h light/dark cycle. This study was carried out in strict accordance with ARRIVE guidelines. The protocols were approved by the Committee on the Ethics of Animal Research of Kyoto Pharmaceutical University (permit numbers: 16-12-034, 17-12-034, and 18-12-034). The number of animals was kept to the minimum necessary for a meaningful interpretation of the data, and animal discomfort was minimized. WT and/or *Trpv4* KO mice were divided into experimental groups according to the random comparison group method.

### Tissue processing and histology

Animals were sacrificed by CO_2_ gas inhalation. Tissues were removed, washed with cold phosphate-buffered saline (PBS), and immersed in 4% paraformaldehyde for 2 h at 4 °C. Tissues were cryoprotected overnight in 20% sucrose solution prior to embedding in optimal cutting temperature compound (Sakura Finetek, Tokyo, Japan) mounting medium. They were then sectioned using a cryostat (Leica Instruments, Nussloch, Germany) at a thickness of 20 µm and thaw-mounted onto Superfrost Plus slides (Matsunami, Osaka, Japan). Immunohistochemical procedures were performed as previously described^13^. Sources of all primary and secondary antibodies, as well as the optimised dilutions, are listed in Supplementary Table 1. TRPV4 immunoreactivity was detected using the fluorescein-conjugated tyramide amplification method (Perkin Elmer Life Sciences, Boston, MA, USA). Cytokeratin 14 and β-catenin were detected using the MOM Immunodetection Kit (FMK-2201; Vector Laboratories, Burlingame, CA, USA). NTPDase2, CAR4, CGRP, 5-HT, PLCβ2, and Ki67 were detected by indirect staining with specific antibodies. P2X2 receptor-ATTO-594 and PLCβ2-Cy5 were detected by direct staining with specific antibodies. The specificity of the TRPV4 antibody was demonstrated by the loss of immunostaining in *Trpv4* KO mice (Fig. 2a).

### Microscopy and image analysis

Sections were viewed using a confocal microscope (A-1R+; Nikon, Tokyo, Japan), and images were captured using Nikon NIS-Elements AR 4.20.00 software. Multiple images in Z-stacks were projected onto a single plane and reconstructed using NIS-Elements AR 4.20.00 software. For quantitative analyses, circumvallate papillae were viewed at 200× magnification using a confocal microscope, and quantitative determinations were made from three random fields for each mouse. For analysis of the TRPV4-, NTPdase2-, PLCβ2-, CAR4-, and P2X2-immunopositive intensities, active areas were measured after interactive thresholding using the NIS-Elements AR 4.20.00 software image analysis system. The relative intensity of the TRPV4 KO was calculated by comparison with the intensity of WT. For analysis, the number of CAR4-immunopositive cells was counted per trench.

### Western blotting

Gastrointestinal tissue preparation was performed as described previously^13^. Lingual epithelial tissue was exfoliated from the tongue by injection of an enzyme cocktail comprising 2.5 mg/mL dispase II (Wako Chemicals, Tokyo, Japan), 1.0 mg/mL collagenase D, and 1.0 mg/mL trypsin inhibitor (Roche Diagnostics, Basel, Switzerland) for 20 min at 25 °C, and then the epithelial tissue was peeled off. Proteins were separated by 7.5% SDS-PAGE and transferred onto polyvinylidene fluoride membranes (Millipore, Bedford, MA, USA) by electroblotting. Three subcellular fractions (cytoplasmic, membrane, and nuclear) of circumvallate papillae were prepared utilizing the subcellular protein fractionation kit (Thermo Fisher Scientific, Rockford, IL, USA) and characterized by western blotting using fraction-specific protein antibodies. The membranes were stained with rabbit anti-β-catenin (1:500; Cell Signaling Technology, Danvers, MA USA) and rabbit anti-β-actin (1:2000; Gene Tex, San Antonio, TX, USA) antibodies (see full-length blot strips in the Supplementary Information). Immunoreactivity was detected by enhanced chemiluminescence (Perkin Elmer Life Sciences, Inc.), and band density was determined using the FUSION solo5 (Vilber Lourmat, Marne-la-Vallée, France).

### Two-bottle tests

Taste preferences were assessed using an ascending concentration series of two-bottle choice tests for 48 h with the following taste compounds: sucrose, sodium glutamate, sodium chloride, denatonium benzoate, and citric acid. In each test series, the mice were trained on two drinking tubes containing deionized water for 1 week, and then a choice between deionized water and the taste compound was given, with each test lasting 96 h. The positions of the two drinking tubes were switched every 24 h. Intake from each tube was obtained by recording the weight of fluid at the beginning and end of each 48-h test. Total fluid intakes were obtained by adding together the intakes from both drinking tubes. Preference ratios were calculated as the intake of each solution divided by total intake and expressed as a percentage.

### Brief-access test

A brief-access test was performed as described previously^44^. In brief, the lick number for distilled water over 10 s was determined, followed by a 15-s inter-presentation interval, and then the taste solution was presented for 10 s, followed by a 5-s water presentation. Because the lick number is affected by differences in motivation to drink solutions among mice, data are expressed as lick ratios as a quantitative index of taste sensitivity and were calculated by dividing the lick number over 10 s for the taste solution by that for distilled water. The number of licks of water used for the lick ratio calculation was recorded preceding the trial with the first sour taste solution (3, 10, 30, and 100 mM citric acid). Data were excluded when mice were unable to complete a series of brief-access tests in each session.

### Reverse transcription and real-time quantitative polymerase chain reaction (PCR) analyses

Total RNA was extracted using a NucleoSpin RNA® XS kit (Macherey-Nagel, Düren, Germany) and a PrimeScript^TM^ RT reagent kit with gDNA Eraser (Takara, Shiga, Japan) and was analysed by quantitative PCR on an ABI 7500 system (Applied Biosystems, Foster City, CA, USA) using SYBR Premix ExTaq II (Takara). Specific primer sets for β-actin, *NTPdase2*, *T1r2*, *T1r3*, *Pkd1l3*, and *CAR4* are listed in Supplementary Table 2. The expression of each target gene was calculated by the comparative Ct method and was normalized to that in WT mice.

### Organoid culture

Organoid culture was performed as described previously^35^. The tongue was isolated, and dispase II (Roche, 1 mg/mL) was injected under the epithelium. After 30 min of incubation at room temperature, the epithelium was peeled away under a dissecting scope, and the circumvallate papilla tissue was isolated. Tissues were incubated with 0.25% trypsin/EDTA for 30 min at 37 °C and centrifuged at 800 × *g* for 5 min. The tissue was suspended in Matrigel (BD Biosciences, Franklin Lakes, NJ, USA), and the suspended tissue was seeded into 12-well culture plates (50 μL Matrigel). After Matrigel polymerization at 37 °C, advanced DMEM/F12 supplemented with 2 mM GlutaMax, 10 mM HEPES, 100 U/mL penicillin, 100 μg/mL streptomycin, and 1 × N2 and 1 × B27 supplements (Thermo Fisher Scientific, Rockford, IL, USA) plus the following growth factors was added to the wells and replaced every 4 days: taste bud organoid medium: Wnt-conditioned medium (50%), R-spondin-conditioned medium (10%), EGF (50 ng/mL, Pepro Tech, Rocky Hill, NJ, USA), and Noggin (100 ng/mL, Pepro Tech).

### Organoid passage

Organoids in Matrigel were collected with cold DPBS (without Ca^2+^/Mg^2+^) and centrifuged at 150 × *g* for 5 min at 4 °C, followed by removal of supernatants including Matrigel. Taste bud organoids were incubated with 0.25% trypsin/EDTA for 30 min at 37 °C and then dissociated into single cells through a 31 G insulin needle. After centrifugation at 800 × *g* for 5 min, cells were re-suspended in Matrigel. Taste bud organoid medium was first changed after 5 days of culturing and then every 3 days. A taste organoid was derived from each WT or *Trpv4* KO mouse. Immunohistochemical staining of taste bud organoids was conducted at 10 days after first passage.

### Statistical analysis

Data are reported as mean ± standard error of the mean and were analysed with GraphPad Prism 6.07 (GraphPad Software, La Jolla, CA, USA). Multiple groups were compared by two-way (genotype and citric acid concentration) ANOVA followed by Bonferroni’s multiple comparison test. Parametric data were tested by Student’s *t*-test. Results were considered statistically significant at *P*-values < 0.05.

## Supplementary information

Supplementary table 1

Supplementary table 2

## Data Availability

The datasets generated during and/or analysed during the current study are available from the corresponding author on reasonable request.
