# Supplementary material for: Transient receptor potential vanilloid 4 mediates sour taste sensing via type III taste cell differentiation
Source: Sci Rep. 2019 Apr 30;9:6686. doi: 10.1038/s41598-019-43254-y (PMC6491610; doi:10.1038/s41598-019-43254-y)
Supplement: Supplementary file 1 — Supplementary table 1 [file 41598_2019_43254_MOESM1_ESM.pdf]

Title page

Title: Transient receptor potential vanilloid 4 mediates sour taste sensing via type III taste cell differentiation

Authors: Kenjiro Matsumoto[1*], Akihiro Ohishi[2], Ken Iwatsuki[3], Kaho Yamazaki[1], Satoko Takayanagi[1], Masahiro Tsuji[1], Eitaro Aihara[4], Daichi Utsumi[1], Takuya Tsukahara[1], Makoto Tominaga[5], Kazuki Nagasawa[2], and Shinichi Kato[1]

Supplementary information

Supplementary Table 1. Primary and secondary antibodies used for immunohistochemistry

Primary antibody	Source<br>*Reference #*	Dilution	Secondary Antibody	Source<br>*Reference #*	Dilution
**Rabbit anti-TRPV4**	Abcam<br>*ab39260*	1/8000	Donkey anti-rabbit IgG biotinylated	Jackson Immuno Research<br>*711066152*	1/400
			TSA Fluorescein Tyramide Reagent Kit	PerkinElmer<br>*SAT701001EA*	1/75
**Sheep anti-ENTPD2 (NTPDase2)**	R&D Systems<br>*AF5797*	1/500	Alexa Fluor® 594 donkey anti-sheep IgG	Invitrogen<br>*A11016*	1/400
**Goat anti-carbonic anhydrase IV/CA4**	R&D Systems<br>*AF2414*	1/500	Alexa Fluor® 488 donkey anti-rabbit IgG	Invitrogen<br>*A11008*	1/400
**Rabbit anti-PLCβ2**	Santa Cruz Biotechnology<br>*sc206*	1/500	Alexa Fluor® 488 donkey anti-rabbit IgG	Invitrogen<br>*A11008*	1/400
		1/100	Cy3® Fast Conjugation Kit	Abcam<br>*ab188287*	1/250
**Sheep anti-CGRP**	Enzo Life Sciences<br>*BML-CA1137*	1/4000	Alexa Fluor® 594 donkey anti-sheep IgG	Invitrogen<br>*A11016*	1/400
**Goat anti-5-HT**	Immunostar<br>*20079*	1/16000	Alexa Fluor® 488 donkey anti-rabbit IgG	Invitrogen<br>*A11008*	1/400
**Rabbit anti-anti-P2X2 receptor-ATTO-594**	Alomone labs<br>*APR-003-AR*	1/200	-	-	-
**Rat anti-Ki67**	Invitrogen<br>*14-5698-80*	1/300	Alexa Fluor® 488 donkey anti-rat IgG	Invitrogen<br>*A21208*	1/400
**Mouse anti-cytokeratin 14**	Abcam<br>*ab7800*	1/100	Mouse on Mouse Fluorescein Kit	Vector<br>*FMK-2201*	1/250
**Mouse anti-β-catenin**	BD biosciences<br>*60153*	1/200	Mouse on Mouse Fluorescein Kit	Vector<br>*FMK-2201*	1/250
**Goat anti-Sonic hedgehog**	R&D Systems<br>*AF464*	1/100	Alexa Fluor® 594 donkey anti-goat IgG	Invitrogen<br>A11058	1/400
