# Supplementary material for: Transient receptor potential vanilloid 4 mediates sour taste sensing via type III taste cell differentiation
Source: Sci Rep. 2019 Apr 30;9:6686. doi: 10.1038/s41598-019-43254-y (PMC6491610; doi:10.1038/s41598-019-43254-y)
Supplement: Supplementary file 2 — Supplementary table 2 [file 41598_2019_43254_MOESM2_ESM.pdf]

Title page

Title: Transient receptor potential vanilloid 4 mediates sour taste sensing via type III taste cell differentiation

Authors: Kenjiro Matsumoto[1*], Akihiro Ohishi[2], Ken Iwatsuki[3], Kaho Yamazaki[1], Satoko Takayanagi[1], Masahiro Tsuji[1], Eitaro Aihara[4], Daichi Utsumi[1], Takuya Tsukahara[1], Makoto Tominaga[5], Kazuki Nagasawa[2], and Shinichi Kato[1]

Supplementary information

Supplementary Table 2. Primers used for quantitative real-time PCR.

Gene		Primer sequences	Product size	Accession #
NTPDase2	F	5'-TTGAGACAACCAGTCCATCTGAA-3'	196 bp	NM_009849.2
	R	5'-AGCAGCACTTGGGTGGAGTA-3'		
T1r2	F	5'- CCAAAGCATCGCCTCCTACTC-3'	119 bp	NM_031873.1
	R	5'- CAGGCTGGCAACTCTTAGAACAC-3'		
T1r3	F	5'-GGCCTTAGGTGGGCATAATAGGA-3'	90 bp	NM_031872.2
	R	5'-AAGGCCTGCAGTGCACAAGA-3'		
Pkd1l3	F	5'-GCCTGTTCAGATGGTTGAAGTG-3'	116 bp	NM_181544
	R	5'-GCTGGTGGCTTGGTCTTTG-3'		
Car4	F	5'-GGTTCAGAGCACAGTATTGATGG-3'	146 bp	NM_007607
	R	5'-CCCTTGTTCACCTTGTCTCCTAC-3'		
β-actin	F	5'-CATCCGTAAAGACCTCTATGCCAAC-3'	171 bp	NM_007393
	R	5'-ATGGAGCCACCGATCCACA-3'		
